# The interplay of research and teaching for better medical care: Balance and dynamic development

**DOI:** 10.3205/zma001863

**Published:** 2026-06-15

**Authors:** Martin R. Fischer

**Affiliations:** 1 LMU University Hospital, LMU Munich, Institute of Medical Education, Munich, Germany

## Editorial

A bon mot attributed to Björn Borg states: “A great tennis player is not automatically a good tennis coach”.

The close connection between research and teaching is regarded as a cornerstone of university education. The unity of research and teaching is considered not only an institutional ideal but is sometimes also viewed as self-evident at the individual level. According to a widespread assumption, those who conduct excellent research also provide high-quality teaching. Likewise, teaching competence is often interpreted as an expression of scientific excellence. But how robust are these assumptions in reality?

Medical education has traditionally operated within the tension field of the so-called Humboldtian triangle of patient care, teaching, and research. This ideal of academic medicine, rooted in Wilhelm von Humboldt’s concept of education, continues to shape the self-understanding of medical faculties and university hospitals today [1]. Unlike many other academic disciplines, scientific knowledge, professional practice, and educational processes are inseparably intertwined in medicine. It is precisely from this close interconnection that medical education research derives its particular relevance.

Medical learning does not occur detached from healthcare delivery but rather in the midst of clinical reality. Students acquire competencies not only in seminar rooms but also in outpatient clinics, hospital wards, and direct contact with patients. The quality of medical education therefore depends substantially on how teaching and learning processes can be designed under the conditions of modern healthcare delivery. Medical education research plays a central role in this context: it investigates how learning succeeds in complex clinical environments, which educational concepts promote sustainable competence development, and how patient safety and quality of care can simultaneously be ensured.

At the same time, the Humboldtian triangle reflects the aspiration to understand research not merely as an institutional mandate but as an integral component of academic education. Medical training should enable future physicians to think scientifically, critically evaluate evidence, and deal reflectively with uncertainty. Consequently, medical education research increasingly addresses questions of scientific reasoning, research-based learning, and the development of an evidence-oriented professional attitude. In doing so, it makes a substantial contribution to strengthening the connection between scientific rigor and clinical practice in medical education.

At the same time, it becomes evident that the relationship between patient care, research, and teaching is by no means free of tension. Economic pressures in healthcare systems, increasing demands for scientific productivity, and limited personnel resources frequently create competing priorities among the three core responsibilities of academic medicine. Teaching in particular is at risk of being structurally overshadowed by clinical duties and research performance. Medical education research highlights these tensions while simultaneously examining the institutional conditions under which high-quality teaching can succeed. It addresses sustainable educational structures, appropriate incentive systems, and forms of academic culture in which teaching is recognized as an equally important component of academic medicine.

This is precisely where its particular significance lies: medical education research does not merely investigate methods of teaching but rather the conditions under which professional identity, clinical judgment, and scientific attitudes emerge. By integrating medical, educational, psychological, and social science perspectives, it makes a central contribution to the further development of academic medicine as a whole.

Against the background of ongoing transformations in healthcare systems, this perspective is gaining increasing importance. Digitalization, the influence of so-called artificial intelligence, interprofessional collaboration, new healthcare structures, and societal expectations regarding patient-centered medicine are changing not only clinical practice but also the requirements for medical education. Medical education research is therefore faced with the task of developing evidence-based answers to the question of how future healthcare professionals can be responsibly educated under changing conditions. The Humboldtian triangle thus remains not merely a historical ideal but also a highly relevant framework for the continued development of academic medicine.

Academic reality presents a more differentiated picture. Visibility in research – measured by external funding, publication output, impact factors, or scientific reputation – does not necessarily correlate with high-quality teaching [2], [3]. Conversely, outstanding teachers are not automatically those who achieve the greatest scientific visibility. This observation is neither new nor surprising, yet it continues to be insufficiently acknowledged within academic systems [4], [5], [6].

Research and teaching follow different logics, incentive systems, and competency profiles. Research rewards innovation, specialization, competition, and international networking. Good teaching, by contrast, requires not only disciplinary expertise but also pedagogical sensitivity, communication skills, reflection, empathy, and a willingness to actively shape learning processes. Whereas scientific success often appears quantitatively measurable, the quality of teaching remains more complex, context-dependent, and frequently less visible and more difficult to assess.

This distinction becomes particularly apparent in medical education. Excellent researchers can inspire students because they convey cutting-edge knowledge and exemplify scientific thinking. At the same time, learners not infrequently encounter lectures or seminars in which disciplinary expertise is not translated into understandable, motivating, or educationally effective teaching. Conversely, there are educators who leave a lasting impression on students, inspire enthusiasm for the discipline, and foster clinical reasoning without themselves being at the center of international research networks.

The question, therefore, is not whether research or teaching is more important. Rather, the question is whether our higher education system truly assigns appropriate value to both domains [7]. Research achievements continue to dominate academic career development. Appointments, promotions, and institutional reputation are still primarily based on scientific metrics. Although teaching performance is increasingly demanded, it is often assessed only as a supplementary rather than an equivalent achievement. The ongoing risk is that teaching is regarded as a “natural side activity” of scientific work rather than as an independent professional competence.

Numerous arguments support greater differentiation. High-quality teaching is not a matter of chance. It is based on pedagogical qualification, curriculum development, feedback culture, and continuous reflection. Likewise, excellent research does not arise solely from talent. Both domains deserve recognition as independent forms of academic excellence [8], [9].

In addition, there is a growing demand for evidence-based teaching in the health professions. Analogous to evidence-based medicine, teaching methods today are expected to rely not primarily on tradition, individual experience, or institutional habit but on empirical educational research. Educators are expected to justify didactic decisions, formulate learning objectives transparently, and evaluate instructional formats based on demonstrable learning outcomes [10]. In this way, teaching itself becomes a scientifically reflected field of practice. This development also underscores that excellent research competence cannot automatically be equated with didactic expertise. Rather, high-quality teaching requires its own scientific competencies in educational research, curriculum development, and evaluation.

This does not, however, imply a separation of research and teaching. On the contrary, the productive integration of both domains remains a central goal of academic medicine. Students benefit from an education that is scientifically grounded, critically reflective, and at the same time learner-centered. What is crucial, however, is the recognition that this connection does not arise automatically but must be actively fostered.

This also requires a cultural change within academic medicine. Teaching competence should be systematically developed, made visible, and institutionally rewarded. Career pathways that give greater consideration to excellent teaching could help reduce the existing asymmetry. At the same time, researchers should be supported in communicating their scientific expertise in educationally effective ways. The question, therefore, is not whether research and teaching are compatible, but under which conditions both qualities can emerge together.

Perhaps the real challenge lies in critically questioning the traditional ideal of “universal academic excellence.” Not every excellent researcher will automatically be an excellent teacher – and not every outstanding teacher will conduct internationally visible research. Recognizing both realities does not weaken academic aspirations; rather, it represents a realistic and appreciative acknowledgment of different academic strengths.

For medical education, this may represent a genuine opportunity: a culture that does not pit research and teaching against one another, yet also does not equate them prematurely. Quality in research and quality in teaching are compatible – not necessarily within a single individual, but above all within a system that takes both equally seriously for the benefit of patients and their families.

## Author’s ORCID

Martin R. Fischer: [0000-0002-5299-5025]

## Competing interests

The author declares that he has no competing interests.
